# A LC-QTOF Method for the Determination of PEGDE Residues in Dermal Fillers

**DOI:** 10.3390/gels9050409

**Published:** 2023-05-13

**Authors:** Giuseppe Alonci, Anna Boussard, Martina Savona, Fabiana Cordella, Gaetano Angelici, Roberto Mocchi, Sabrina Sommatis, Damiano Monticelli

**Affiliations:** 1 Matex Lab Switzerland SA, 1228 Plan-les-Ouates, Switzerland; 2 Dipartimento di Chimica e Chimica Industriale, Università di Pisa, Via Moruzzi 13, 56124 Pisa, Italy; 3 UB-CARE S.r.l., Spin-Off University of Pavia, Via della Scienza 12, 27100 Pavia, Italy; 4 Department of Science and High Technology, University of Insubria, 22100 Como, Italy

**Keywords:** hydrogels, crosslinkers, QTOF, HPLC, validation, high-resolution mass spectrometry, dermal fillers, hyaluronic acid

## Abstract

Hyaluronic acid is one of the most important ingredients in dermal fillers, where it is often cross-linked to gain more favorable rheological properties and to improve the implant duration. Poly(ethylene glycol) diglycidyl ether (PEGDE) has been recently introduced as a crosslinker because of its very similar chemical reactivity with the most-used crosslinker BDDE, while giving special rheological properties. Monitoring the amount of the crosslinker residues in the final device is always necessary, but in the case of PEGDE, no methods are available in literature. Here, we present an HPLC-QTOF method, validated according to the guidelines of the International Council on Harmonization, which enables the efficient routine examination of the PEGDE content in HA hydrogels.

## 1. Introduction

Hyaluronic acid is a natural polysaccharide that is widely employed and studied in biomedicine and is composed of repeating dimers of D-glucuronic acid and D-N-acetylglucosamine, linked via alternating β-1,4 and β-1,3 glycosidic bonds. It is highly abundant in most living organisms and is one of the main components of the skin, where it gives elasticity, support, and hydration [1,2].

In aesthetic medicine, hyaluronic acid is the mainstay in dermal fillers, because of the limited allergic reactivity, the availability of a remedy to treat overcorrections or vascular complications (hyaluronidase), and for the flexibility in formulation and chemical modification, that allows a wide selection of products fulfilling different aesthetic goals [3,4].

Physiologically, hyaluronic acid is part of a complex homeostatic balance. Non-crosslinked HA is quickly degraded in the body by hyaluronidases, a class of enzymes naturally present in the skin. To reduce this effect and to improve the rheological properties, HA dermal fillers are often crosslinked to increase the residence time after implantation up to 9–12 months [5]. Additionally, while pure hyaluronic acid is a viscous liquid that gives low volumization when used as a filler, crosslinked HA hydrogels behave as viscoelastic solids with more effective aesthetic outcomes [6,7,8].

During the crosslinking process, a bifunctional molecule is employed to bind the linear HA chains, forming a three-dimensional structure that is capable of supporting the mechanical stress of the tissues, but also hinders the action of hyaluronidase, thus reducing the degradation kinetic [9,10]. The properties of hyaluronic acid fillers depend on the crosslinking degree—with a high crosslinking density being related to higher viscoelastic modulus and a slower degradation rate. However, attention must be paid to avoid a too-high extrusion force, poor biointegration, foreign-body reactions, or other side effects due to the presence of crosslinker residues [11].

Several molecules have been employed as crosslinkers [12,13,14,15], such as divinyl sulfone (DVS), 1- ethyl-3-(3 dimethylaminopropyl) carbodiimide (EDC), 1,2,7,8-diepoxyoctane, and glutaraldehyde (GA), with 1,4-butanediol diglycidyl ether (BDDE) being the most commonly used in the dermal filler industry, because of its biodegradability and high biocompatibility [16,17]. In recent years, poly(ethylene glycol) diglycidyl ether (PEGDE) has been introduced, because it has a similar chemical reactivity to BDDE [5,18], but at the same time allows special rheological properties and high cohesivity thanks to the higher hydrophilicity and the longer molecular chain [7].

Although both BDDE and PEGDE are demonstrated to be safe, their presence and quantity must be monitored in the final product. In the case of BDDE, a concentration lower than 2 ppm in the final product is generally considered to be safe [19], and a similar value can be accepted also for PEGDE [20,21].

Several analytical methods are available for the determination of BDDE, such as GC-MS, LC-MS, or spectrofluorimetry [22,23,24,25].

However, no methods for the quantification of PEGDE have been published to our knowledge, even though PEGDE became familiar as a crosslinker, and so a precise and accurate analytical method is necessary to monitor its concentration in HA dermal fillers.

From an analytical point of view, this task is challenging not just because of the complexity of the hydrogel matrix, but also because PEGDE is generally employed as a mixture of different oligomers, which makes it more difficult to analyze with respect to BDDE [23].

Quadrupole time-of-flight mass spectrometry (QTOF) is a technique in high-resolution mass spectrometry that is commonly used to study the composition of complex mixtures, in extractables and leachables studies, and, more generally, in targeted and untargeted analysis. Here, we describe an HPLC-QTOF (high-performance liquid chromatography coupled with quadrupole time-of-flight mass spectrometry) method that allows a precise and reliable quantification of PEGDE in HA dermal fillers at low concentration level and with minimal sample preparation.

## 2. Results and Discussion

### 2.1. PEGDE Reference

PEGDE employed in the crosslinking of hyaluronic acid hydrogels is a mixture of different oligomers with an average molecular weight of 500 ± 50 Da. However, PEGDE is not yet available as a certified reference material, and thus the purity of the reference used in this study had to be assessed. We have decided to perform this task optimizing an LC-QTOF method. However, the possible fragmentation in QTOF could be not efficient, with pseudomolecular ions (mainly the M + NH_4_^+^ ion) and their +1 as the only observable ions. The use of additional techniques based on different principles, i.e., Py-GC-MS and NMR, allows a more reliable identification and purity determination of the reference material, while the use of a single technique can sometimes miss some impurities.

**Figure 1:**
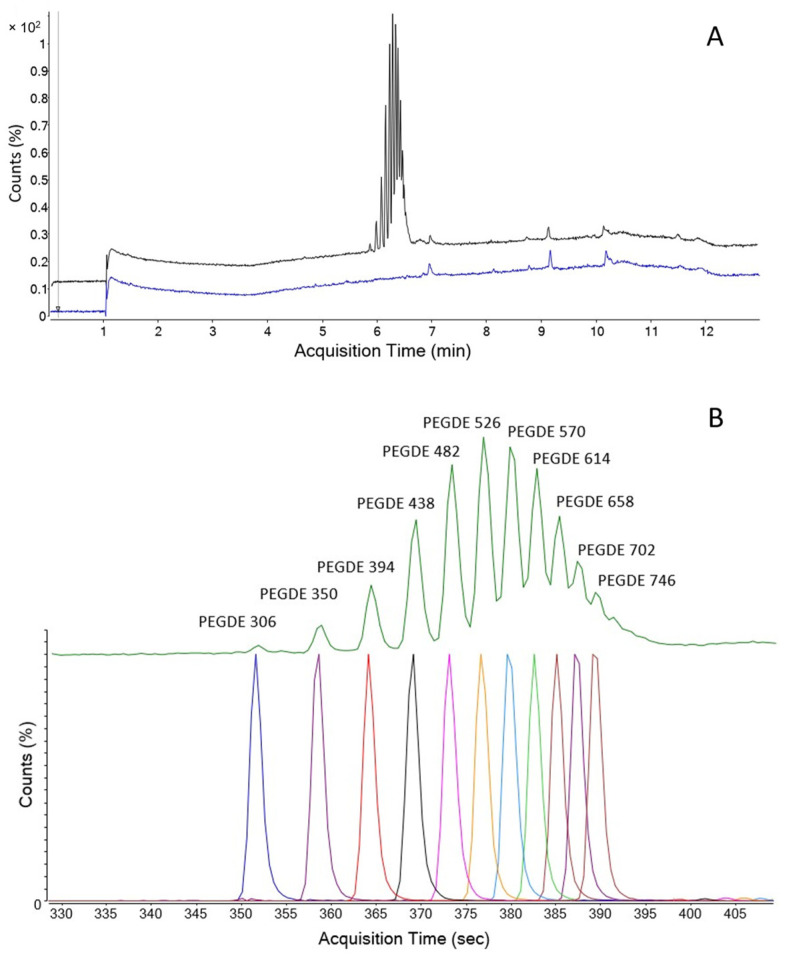
(**A**). Overlayed TIC of PEGDE from BOC Science (black) and Blank solvent (blue). (**B**). Zoom on the PEGDE region of the TIC (top green line) and EIC of 11 PEGDE M + NH_4_^+^ ions.

A 43 µg/mL solution of PEGDE in acetonitrile has been prepared and analyzed via LC-QTOF according to the method reported in Paragraph S1 of the Supplementary Information. The total ion chromatogram (TIC) and extracted ion chromatogram (EIC) are reported in Figure 1 and show only the presence of the peaks corresponding to the expected PEGDE oligomers. The overlayed PEGDE and blank chromatograms (TIC in Figure 1A) show that no other extra peak was identified.

**Figure 2:**
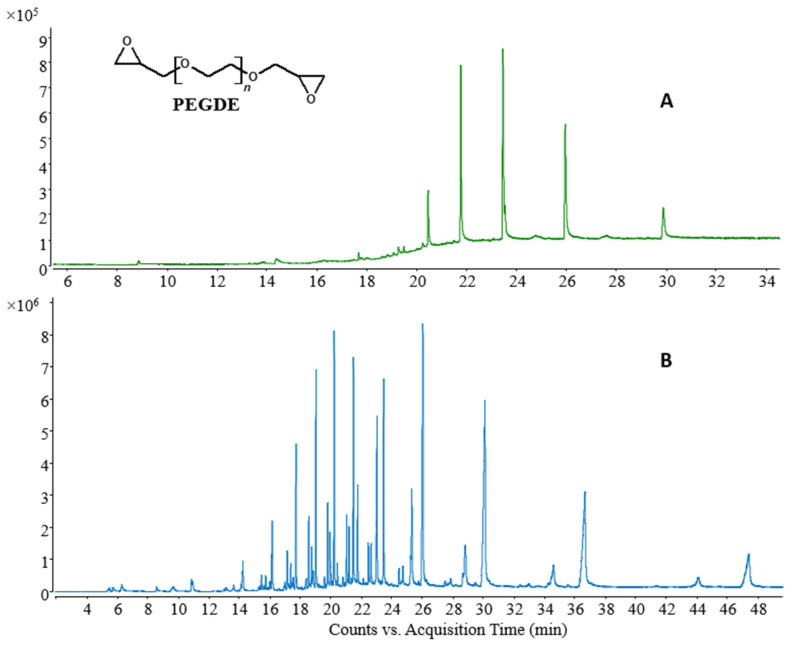
Py-GC/MS analysis of BOC Sciences PEGDE; (**A**) first shot: Split 1:100, T = 300 °C; (**B**) second shot: Split 1:200; T = 600 °C.

PEGDE was also analyzed through pyrolysis-GC/MS (Py-GC/MS) by using a multi-shot pyrolyzer. The first shot was performed at 300 °C, while the second one was performed at 600 °C, as reported in Figure 2. The temperatures chosen for the double-shot pyrolysis are based on standard conditions used for polymer analysis, as it is generally acknowledged that no reactions occur at 300 °C, except for the dehydration reaction, so it is possible to obtain an easy-to-interpret pyrogram that allows to identify any small polluting organic molecules, while decomposition products of the PEGDE oligomers can be observed at 600 °C.

The mass spectra of selected peaks of the pyrogram in the first and second shot are reported in Figure S1. All the peaks have similar mass spectra, no impurities or unexpected peaks were identified, the *m*/*z* ratios associated to each chromatographic peaks are the same both for the first and second shot, and all the ions can be associated to the fragmentation of the PEGDE chain, as explained in Figure 2 and Figure S3 (Supporting Information).

**Figure 3:**
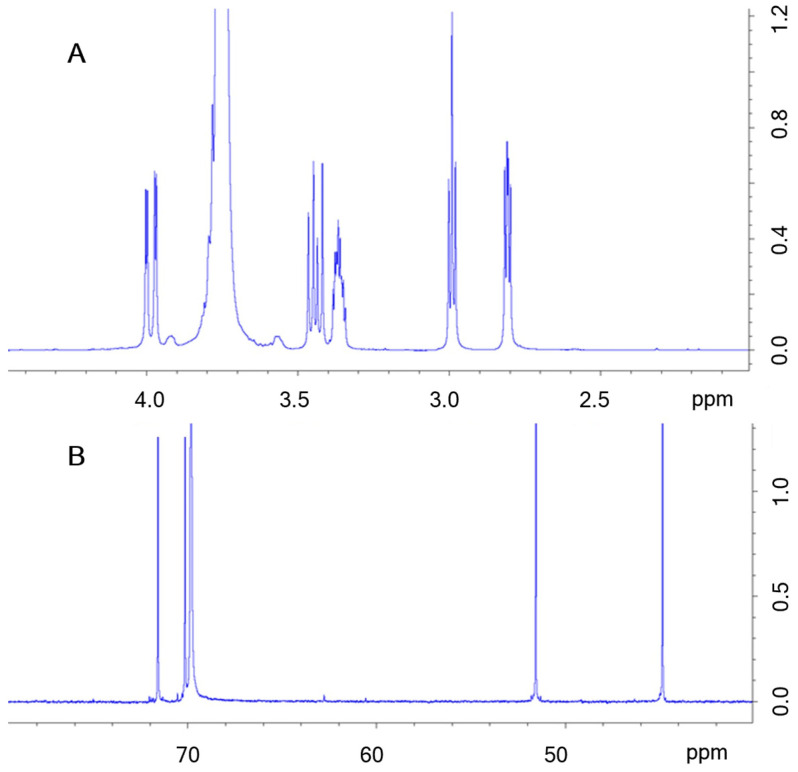
Magnification of ^1^H (**A**) and ^13^C (**B**) NMR spectra in the 2–4.5 ppm and 60–80 ppm ranges, respectively. Chemical shift ranges were selected where PEGDE signals were registered.

Finally, ^1^H and ^13^C NMR spectra were also recorded to further support the purity of the product. Both spectra conform to the expected PEGDE signals and, for ^1^H spectrum, multiplicity (see Figure 3).

The two spectra are compliant with the expected molecular structure of PEGDE and no extra peaks have been observed.

### 2.2. Target Selection

Eight different oligomers with molecular weight ranging from 394 to 702 were selected as analytical targets. Table 1 contains the monoisotopic mass of the eight PEGDE oligomers investigated in this study and of their most common ions.

**Figure 4:**
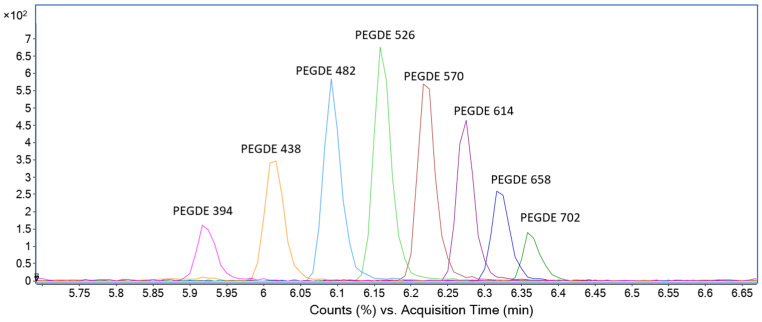
Overlayed EIC of the selected PEGDE oligomers from BOC Science. PEGDE is a mixture of different oligomers; 8 oligomers were selected for the determination.

In TOF spectrometry, being a method based on ESI, the fragmentation is usually very low, and the most important peak is the pseudomolecular ion. The instrument response in the calibration curve and in the analysis of the samples was the sum of the counts of the M + NH_4_^+^ ion of each oligomer (EIC of all the ions are shown in Figure 4). Ions M + NH_4_^+^ +1 were selected in ratio 1/30 as qualifiers, the 1/30 ratio coming from the calculated isotopic abundance. A mass error of ±5 ppm was considered acceptable.

### 2.3. Matrix Effect

**Figure 5:**
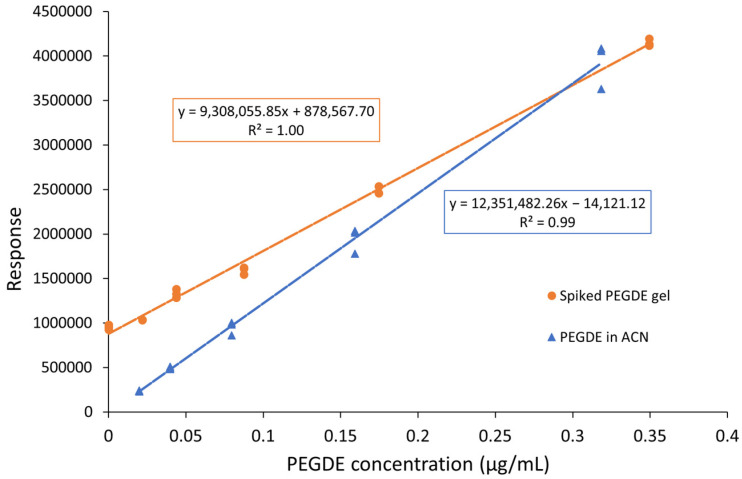
Comparison of the calibration curve of PEGDE in ACN (blue) and of standard PEGDE addition on a PEGDE-crosslinked HA hydrogel (orange).

Hydrogels are very complex materials, and they may lead to a significant matrix effect. Additionally, the presence of other residues such as non-harmful hydrolysis products can interfere with the analysis. To investigate these variables, the calibration curve obtained by fitting with a linear regression model the response of five PEGDE reference solutions in acetonitrile (ACN_R1-ACN_R5), analyzed in triplicate with the method reported in Section 4.5.3, was compared with a curve similarly obtained by spiking a PG-HA hydrogel with known amounts of PEGDE (PEGDE_SP1-PEGDE_SP5) and then extracting the sample with acetonitrile as described in Section 4.4. The extraction with acetonitrile allows to precipitate the insoluble hyaluronic acid, that can be then removed by centrifugation, while the supernatant contains the dissolved residues. The results of these evaluations are shown in Figure 5. 

The slope of the two curves is significantly different, demonstrating a considerable matrix effect that must be addressed, and further method optimalizations were needed.

**Figure 6:**
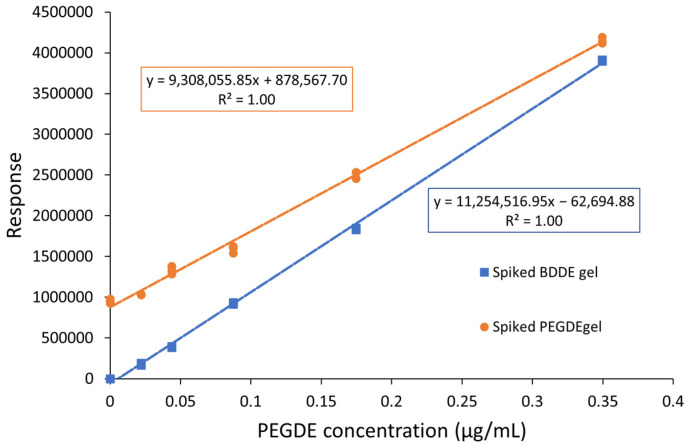
Comparison of calibration curve of spiked BDDE gel (blue) and PEGDE gel (orange).

The method of standard addition can be employed when a significant matrix interference is observed, but it is not practical to be used in routine analysis as it is time consuming and requires a high volume of sample. To verify the possibility of using external calibration, a third calibration curve was prepared by analyzing in triplicate samples prepared by spiking a 26 mg/mL BDDE-crosslinked HA hydrogel (BD-HA) to simulate the hydrogel matrix from the same manufacturer. The same amount (approximately 500 mg) of gel was weighed for the two calibration curves’ comparison. The calibration curves of spiked BDDE gels and PEGDE gels are compared in Figure 6. The difference in slope for the two calibration curves is lower than 20%, although statistically different.

To confirm that the error in the concentration determination is acceptable, the PEGDE concentration in the pristine PG-HA gel was calculated according to the standard addition method and from the calibration curve obtained with the spiking of the BD-HA hydrogel.

In the standard addition method, the concentration of the analyte is calculated by measuring the response of the sample and of the sample spiked with known amounts of analyte, then interpolating the data with the least-squares method, and finally by calculating the x-axis intercept with Equation (1).

(1) $$[PEGDE]=\frac{Intecept}{Slope}$$

In this study, the responses of the blank sample and of five spiked samples at increasing concentrations (samples PEGDE_SP1 to PEGDE_SP5, prepared as described in Section 4.3) were measured in triplicate and analyzed as previously described, and the interpolation curve is reported in Figure 5 and Figure 6. From the equation of the regression curve, it is possible to calculate the initial PEGDE concentration as:

(2) $$[PEGDE]=\frac{878,567.70}{9,308,055.85}=0.094\;\mu g/mL$$

The concentration was determined also according to the calibration curve of the spiked BDDE gel (BDDE_SP1 to BDDE_SP5, prepared as described in Section 4.4). The equation of the calibration curve is reported in Figure 6, and the concentration of the analyte in the PG_HA gel sample is:

(3) $$[PEGDE]=\frac{Response+Intercept}{Slope}=\frac{952,960.70+62,694.88}{11,254,516.95}=0.090\;\mu g/mL$$

The two results differ by less than 5%, demonstrating that an external calibration with a spiked BD-HA hydrogel gives accurate results.

Based on these results, we have then decided to validate the method for determination of PEGDE in HA gels with the calibration curve prepared in spiked BDDE-HA gel.

### 2.4. Method Validation

#### 2.4.1. Linearity and Range

**Figure 7:**
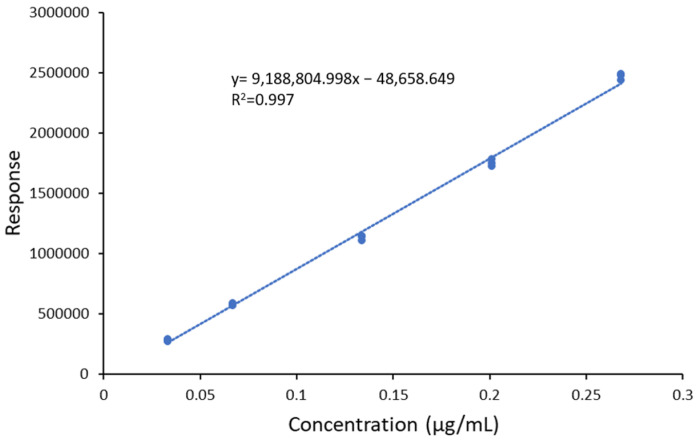
Calibration curve of PEGDE reference solutions.

To verify the linearity and linear range of the method, a new calibration curve was obtained by measuring the response of five BD-HA calibration solutions at five concentration levels (BDD_CAL_1-BDD_CAL_5), analyzed in triplicate (Figure 7). The test was performed on a different set of measures from the one reported in Section 2.3, as the new sequence included all the experiments needed for the full validation of the method.

The fitting was performed with the software Agilent MassHunter Quantitative Analysis, version 10.2 (Agilent Technologies, Santa Clara, CA, USA), using a linear regression model. 

R^2^ was found to be higher than 0.99, and the accuracy was always in the ±20% range of the nominal value of the reference sample. The curve is thus considered linear at least in the calibration range 0.033–0.268 µg/mL, which corresponds to a concentration of 66–536 ppb in the hydrogel. 

#### 2.4.2. LOD and LOQ

For the determination of the LOD, a blank matrix BD_HA was spiked at [PEGDE] = 0.017 µg/mL (corresponding to 34 ppb in the gel) and analyzed in triplicate. The signal-to-noise ratio was investigated for each of the selected ions. The results, summarized in Table 2, demonstrate that the SNR is always higher than 3 at this concentration; if the sum of the eight PEGDE oligomers is to be determined, a safe LOD of 0.017 µg/mL may be assumed. If only the PEGDE 570 ion is used for quantification, e.g., when very low concentrations are targeted and low abundance oligomers are undetected, the LOD can be calculated to be 4 ppb (SNR = 3).

To investigate the LOQ, five samples were prepared by spiking the BD_HA matrix at [PEGDE] = 0.034 µg/mL (corresponding to 68 ppb in the gel) and calculating the SNR again. The results are summarized in Table 3 and show that the SNR is higher than 10 for all the oligomers. If only PEGDE 570 is considered for the quantification, then the LOQ is 15 ppb (SNR = 10).

#### 2.4.3. Precision

Precision was evaluated by 15 determinations covering the analytical range for the procedure, with three replicate injections in five concentration levels, following the indications of ICH Q2 (R1).

The precision on every concentration level was assessed by the RSD, which was calculated according to Equation (4).

(4) $$RSD=\frac{SD}{Average\;response\;of\;PEGDE}\times 100$$

The experiment was repeated on two different days by two different operators.

Results are summarized in Table 4 and show that the RSD is always lower than 10% at all concentration levels.

#### 2.4.4. Accuracy

Accuracy is addressed in the validation of an analytical method to show that the measured value is close the known value. Generally, a blank matrix is spiked with a known amount of analyte, and accuracy is determined simply as the ratio between the measured value and known spiked concentration. However, as BDDE hydrogels have been used to build the calibration curve, they cannot be used a blank matrix to evaluate the accuracy, and a true blank matrix of a PEGDE-hydrogel is not available, as any PEGDE hydrogel inevitably contains some PEGDE residues. Thus, in this study, accuracy is evaluated by comparing the measured PEGDE concentration in the spiked sample with the sum of the known concentration of PEGDE in the standard solution and the measured PEGDE concentration in the unspiked sample.

The test was performed at three spike reference levels (LOQ = 67 ppb, 134 ppb, 268 ppb). PEGDE concentration was measured by comparing the sample response to a calibration curve obtained from samples BDD-CAL-1 to BDD-CAL-5. The accuracy was determined as recovery percentage according to ICH Q2R1 and calculated according to the Equation (5):

(5) $$Recovery\left[\%\right]=\frac{{\left[PEGDE\right]}_{spiked\;sample}}{{\left[PEGDE\right]}_{added\;std}+{\left[PEGDE\right]}_{sample}}\times 100$$

where [PEGDE]_spiked sample_ is the measured concentration of PEGDE in the sample after spiking, [PEGDE]_added std_ is the concentration of the added standard, while [PEGDE]_sample_ is the PEGDE concentration in the unspiked sample.

The results for the [PEGDE]_sample_ determination are summarized in Table 5.

The average result is then used in Equation (6) to calculate the recovery.

The measured PEGDE concentration in the sample and in the spiked sample was normalized to 0.5000 g.

(6) $${\left[PEGDE\right]}_{normalized}={\left[PEGDE\right]}_{measured}\frac{0.5000}{sample\;weight\;(g)}$$

The results of the accuracy evaluation are summarized in Table 6.

#### 2.4.5. Specificity

**Figure 8:**
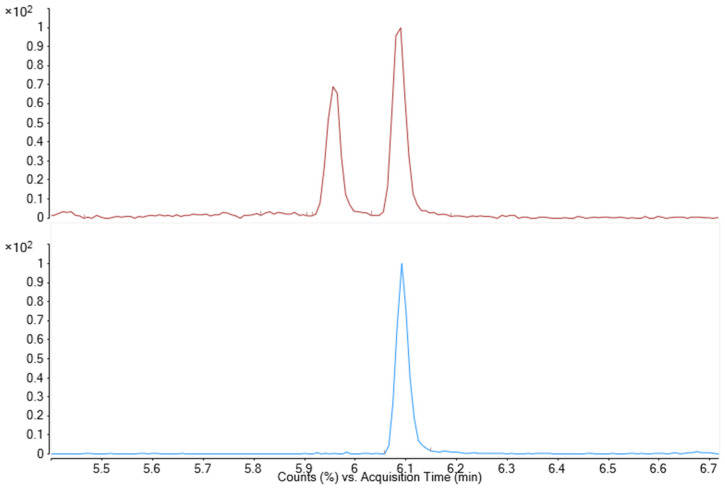
EIC of PEGDE 482 in reference solution (blue, RT = 6.09 min) and in PEGDE gel (red, RT = 6.09 min). In PEGDE gel, other impurity was observed and separated from PEGDE chromatographically. Only the EIC of PEGDE 482 is shown for clarity.

HPLC-QTOF is a highly specific technique that allows to unequivocally identify a compound from its retention time, the high-resolution mass of its ions and from the isotopic abundance, that in this study is considered by using the M + 1 ion as a qualifier only if its abundance was compliant with the 1/30 theoretical isotopic abundance. However, interfering ions were observed with the same exact mass of PEGDE, probably coming from the fragmentation of other impurities present in the hydrogel (such as the subproducts of PEGDE hydrolysis in water). These impurities are separated chromatographically and do not interfere with the measure, as shown in Figure 8. No signal was observed in blank samples (non-spiked BD-HA hydrogels). The identity of each ion in the real-life sample (PEGDE gel) is thus confirmed by its high-resolution mass (±5 ppm), the presence of the +1 ion with the correct isotopic abundance, and by the comparison of retention time with the reference standard (±0.03 min).

## 3. Conclusions

An LC-QTOF method was developed for the determination of PEGDE residues in HA gels that requires only minimal sample preparation.

A significant matrix effect was observed, so a BDDE-HA gel was used as a blank matrix for the preparation of the calibration curve. The linearity, range, accuracy, precision, and specificity of the method were investigated, and the results fulfill the requirements of ICH Q2 (R1). The proposed method allows to quantify a concentration of PEGDE residues as low as 68 ppb, which is well below the accepted threshold of 2 ppm and is thus suitable to be used in R&D and quality control laboratories.

## 4. Materials and Methods

### 4.1. Standards and Reagents

Poly(ethylene glycol) diglycidyl ether (PEGDE) was purchased from BOC Science (New York, NY, USA, CoA available in Paragraph S3 of the Supporting Information) and used as received. Acetonitrile for LC-MS was purchased from VWR (Briare, France). BDDE-crosslinked 26 mg/mL hyaluronic acid hydrogel (BD_HA) and PEGDE-crosslinked 26 mg/mL hyaluronic acid hydrogel (PG_HA) were provided by Matex Lab S.p.A (Brindisi, Italy). 

### 4.2. Py-GC-MS

Py-GC/MS analysis was performed using a multi-shot pyrolyzer EGA/PY-3030D (Frontier Lab, Saikon, Japan) coupled to an 8890 gas chromatograph, combined with a 5977B mass selective single quadrupole mass spectrometer detector (Agilent Technologies, Santa Clara, CA, USA). The parameters used for the mass spectrometer unit (MS) are: electron impact ionization (EI 70 eV) in positive mode; ion source temperature: 230 °C; scan range: 35–600 *m*/*z*; interface temperature: 280 °C. The analysis was performed in double shot mode at two different temperatures for subsequent analyses of the same sample: 300 °C for the first shot and 600 °C for the second shot. A total of 1.25 mg of PEGDE were directly weighted in a deactivated stainless-steel cup and inserted in the furnace. 

The pyrolysis products were separated with an HP-5MS capillary column (95% dimethyl-5% diphenyl-polysiloxane; 30 m × 0.25 mm, film thickness 0.25 µm; Agilent Technologies, Santa Clara, CA, USA). The Py-GC interface was set at 280 °C and the GC injector was operated in split mode: 1:100 for the first shot and 1:200 for the second shot. The chromatographic conditions for the analysis were: 40 °C for 6 min, 20 °C/min up to 310 °C, held for 40 min. Helium (He, purity 99.9995%) was used as gas carrier, with a constant flow of 1.2 mL/min.

### 4.3. Nucler Magnetic Resonance Spectra

The ^1^H and ^13^C NMR spectra were acquired on a Brucker 400 MHz Advance NMR spectrometer equipped with a 5 mm PABBO probe. The zg30 sequence from the TopSpin library was used to acquire ^1^H spectra, whereas ^13^C spectra were acquired by the zgig sequence. The ^1^H spectra were acquired accumulating 8 scans with a recycle delay (D1) time of 4 s, whereas ^13^C spectra acquisition required a D1 of 15 s and the accumulation of 1024 scans. These delays ensured full relaxation and, accordingly, quantitative signals. The PEGDE sample was simply diluted 1:2 with deuterium oxide prior to analysis. Tetramethylsilane was used for spectra calibration.

### 4.4. Matrix Effect

#### 4.4.1. PEGDE Reference Solutions (Acetonitrile)

PEGDE solutions were prepared in acetonitrile, at 5 different concentration levels, by dilution of a PEGDE stock solution (0.398 µg/mL) according to Table 7 and then analyzed according to the procedure described in Section 4.5.3.

#### 4.4.2. Spiked HA-Hydrogels

BDDE (Table 8) and PEGDE (Table 9) hydrogels were weighted into a 10 mL centrifuge tube and then were spiked with a PEGDE stock solution (0.437 µg/mL in acetonitrile). The solution was sonicated for 5 min and centrifugated on 4500 rpm for 5 min. The supernatant was then analyzed according to the procedure described in Section 4.5.3.

### 4.5. Method Validation

#### 4.5.1. BD-HA Calibration Solution

Calibration solutions were prepared by BDDE gel spiking with PEGDE solution at 5 different concentration levels. PEGDE stock solution in acetonitrile (0.335 µg/mL) was used for spiking (Table 10). Hyaluronic acid of the gel was extracted by sonication (5 min) and centrifugation (4500 rpm/5 min). The supernatant was used for the sample solution and examined according to the procedure described in Section 4.5.3.

#### 4.5.2. PG-HA Spiked Samples

Calibration solutions were prepared by PEGDE gel spiking with PEGDE solution at 3 different concentration levels. PEGDE stock solution in acetonitrile (0.335 µg/mL) was used for spiking (Table 11). Hyaluronic acid of the gel was extracted by sonication (5 min) and centrifugation (4500 rpm/5 min). The supernatant was used for sample solution and examined according to the procedure described in Section 4.5.3.

#### 4.5.3. Sample Preparation and Analytical Conditions

Approximately 0.5 g of sample were exactly weighted in a test tube. A total of 1 mL of acetonitrile was added to the sample, which was then sonicated for 5 min and then centrifugated at 4500 rpm for 5 min. The supernatant was then transferred to a 1 mL LC-MS vial and analyzed on an Agilent 1260 Infinity II HPLC system coupled to an Agilent 6530 Q-TOF mass spectrometer. Chromatographic separation was performed using Agilent InfinityLab Poroshell 120EC C18 column (150 × 3.0 mm, 2.7 µm). The QTOF was tuned according to Agilent instructions before each sequence and acquisition was performed in positive mode at 2 spectra/s from 100 to 1000 m/z. All method details and parameters are reported in Paragraph S2 of the Supporting Information.

## Figures and Tables

**Table 1:** The monoisotopic mass of the eight PEGDE oligomers and of their most common ions which were investigated for method development and validation. The sum of the response of the M + NH_4_^+^ ion of each oligomer was used as the analytical signal.

Name	Formula	M	M + H^+^	Quantifiers (M + NH_4_^+^)	Qualifiers (M + NH_4_^+^ 1^+^)
PEGDE 394	C_18_H_34_O_9_	394.2203	395.2275	412.2541	413.2568
PEGDE 438	C_20_H_38_O_10_	438.2465	439.2538	456.2804	457.2831
PEGDE 482	C_22_H_42_O_11_	482.2727	483.2800	500.3066	501.3135
PEGDE 526	C_24_H_46_O_12_	526.2989	527.3062	544.3328	545.3378
PEGDE 570	C_26_H_50_O_13_	570.3251	571.3324	588.3595	589.3612
PEGDE 614	C_28_H_54_O_14_	614.3514	615.3586	632.3852	633.3897
PEGDE 658	C_30_H_58_O_15_	658.3776	659.3848	676.4114	677.4137
PEGDE 702	C_32_H_62_O_16_	702.4038	703.4110	720.4377	721.4410

**Table 2:** Average SNR in samples spiked at the LOD (0.017 µg/mL).

Name	Retention Time	Average Signal-to-Noise
PEGDE 394	5.92	4.1
PEGDE 438	6.02	9.6
PEGDE 482	6.09	15.9
PEGDE 526	6.17	22.5
PEGDE 570	6.22	24.5
PEGDE 614	6.27	19.3
PEGDE 658	6.32	13.8
PEGDE 702	6.38	7.4

**Table 3:** Average SNR in samples spiked at the LOQ (0.034 µg/mL).

Name	Retention Time	Average Signal-to-Noise
PEGDE 394	5.92	13.46
PEGDE 438	6.02	22.34
PEGDE 482	6.09	43.02
PEGDE 526	6.17	33.98
PEGDE 570	6.22	46.92
PEGDE 614	6.27	37.26
PEGDE 658	6.32	25.38
PEGDE 702	6.38	15.06

**Table 4:** Precision evaluation by measuring the RSD of repeated measures on spiked BD-HA hydrogel.

Samples	Concentration (µg/mL)	RSD (%)
Sequence 1		
BD-HA-SP1-S1 (LOQ)	0.022	6.76
BD-HA-SP2-S1	0.044	2.11
BD-HA-SP3-S1	0.087	1.37
BD-HA-SP4-S1	0.175	0.57
BD-HA-SP5-S1	0.349	0.26
Sequence 2		
BD-HA-SP1-S2 (LOQ)	0.033	2.43
BD-HA-SP2-S2	0.067	1.61
BD-HA-SP3-S2	0.134	1.71
BD-HA-SP4-S2	0.201	1.72
BD-HA-SP5-S2	0.268	1.16

**Table 5:** Measured PEGDE concentration in sample. The analysis was performed in triplicate injections.

Sample	Weighing	[PEGDE]_sample_ (ppb)
PG-HA	1	126
	2	127
	3	121
Average		125
SD		3.2

**Table 6:** Result of the recovery evaluation. Measurement performed at 3 different concentrations by triplicate injections. The recovery is the ratio of the measured PEGDE concentration in spiked sample solution and the sum of the known concentration of PEGDE in reference solution and measured PEGDE concentration in sample.

Sample	[PEGDE]_ref_ (ppb)	Injection	[PEGDE]_sample_ (ppb)	[PEGDE]_spiked_ (ppb)	Recovery (%)
PG-HA-1	67	1	125	149	78
		2		147	77
		3		145	75
PG-HA-2	134	1		232	90
		2		228	88
		3		225	87
PG-HA-3	268	1		400	102
		2		409	104
		3		407	104

**Table 7:** Preparation of PEGDE solutions in ACN. The solution prepared at 5 concentration levels diluted from PEGDE stock solution (0.398 µg/mL).

Reference	PEGDE Stock Solution (µL)	ACN (µL)	[PEGDE] (µg/mL)
ACN_R1	50	950	0.020
ACN_R2	100	900	0.040
ACN_R3	200	800	0.080
ACN_R4	400	600	0.159
ACN_R5	800	200	0.318

**Table 8:** Preparation of spiked BDDE gel. The concentration of PEGDE stock solution is 0.437 µg/mL in acetonitrile.

Reference	BD-HA (g)	PEGDE Stock Solution (µL)	ACN (µL)	[PEGDE] (µg/mL)
BDDE_SP1	0.3360	50	950	0.022
BDDE_SP2	0.5714	100	900	0.044
BDDE_SP3	0.5095	200	800	0.087
BDDE_SP4	0.5223	400	600	0.175
BDDE_SP5	0.5239	800	200	0.349

**Table 9:** Preparation of spiked PEGDE gel. The concentration of PEGDE stock solution is 0.437 µg/mL in acetonitrile.

Reference	PG-HA (g)	PEGDE Stock Solution (µL)	ACN (µL)	[PEGDE] (µg/mL)
PEGDE_SP1	0.4523	50	950	0.022
PEGDE_SP2	0.5347	100	900	0.044
PEGDE_SP3	0.3986	200	800	0.087
PEGDE_SP4	0.4565	400	600	0.175
PEGDE_SP5	0.4852	800	200	0.349

**Table 10:** Preparation of calibration solutions. Calibration solutions were prepared by BDDE gel spiking with PEGDE solution at 5 different concentration levels. Concentration is referred to 0.5 g theoretical mass and later corrected according to Equation (3).

Reference	BD-HA (g)	PEGDE Stock Solution (µL)	ACN (µL)	[PEGDE] (ppb)
BDD-CAL-1	0.5667	100	900	67
BDD-CAL-2	0.5411	200	800	134
BDD-CAL-3	0.5535	400	600	268
BDD-CAL-4	0.5138	600	400	402
BDD-CAL-5	0.5001	800	200	536

**Table 11:** Preparation of spiked sample solutions. Spiked sample solutions were prepared by PEGDE (PG-HA) gel spiking with PEGDE solution at 5 different concentration levels.

Reference	PG-HA (g)	PEGDE Stock Solution (µL)	ACN (µL)	[PEGDE] Ref (ppb)
PG-SP-1	0.4956	100	900	68
PG-SP-2	0.5005	200	800	134
PG-SP-3	0.4315	400	600	268

## Data Availability

The data presented in this study are available on request from the corresponding author.

## Supplementary Materials

The following supporting information can be downloaded at: https://www.mdpi.com/article/10.3390/gels9050409/s1, Supporting information, including details of the chromatographic methods, are available to download.
