# Supplementary material for: A LC-QTOF Method for the Determination of PEGDE Residues in Dermal Fillers
Source: Gels. 2023 May 13;9(5):409. doi: 10.3390/gels9050409 (PMC10217355; doi:10.3390/gels9050409)

gels

MDPI

Protocol

A LC-QTOF Method for the Determination of PEGDE Residues in Dermal Fillers

Giuseppe Alonci [1,*], Anna Boussard [1], Martina Savona [1], Fabiana Cordella [2], Gaetano Angelici [2], Roberto Mocchi [3], Sabrina Sommatis [3] and Damiano Monticelli [4]

S1. PEGDE qualification method details

The HPLC-QTOF measures were performed on an Agilent 1260 Infinity II HPLC system coupled to an Agilent 6530 Q-TOF mass spectrometer. Chromatographic separation was performed using Agilent InfinityLab Poroshell 120EC C18 column (150 x 3.0 mm, 2.7µm).

Table S1. HPLC-QTOF method details for qualification of PEGDE reference material.

<table>
<tr><th colspan="2">LC</th></tr>
<tr><td>Gradient</td><td>
<table>
<tr><th>Time
(min)</th><th>A*
(%)</th><th>B **
(%)</th><th>Flow
(mL/min)</th></tr>
<tr><td>0.00</td><td>1.0</td><td>99.0</td><td>0.600</td></tr>
<tr><td>1.00</td><td>1.0</td><td>99.0</td><td>0.600</td></tr>
<tr><td>7.00</td><td>80.0</td><td>20.0</td><td>0.600</td></tr>
<tr><td>8.00</td><td>90.0</td><td>10.0</td><td>0.600</td></tr>
<tr><td>9.00</td><td>100.0</td><td>0</td><td>0.600</td></tr>
<tr><td>10.00</td><td>100.0</td><td>0</td><td>0.600</td></tr>
<tr><td>11.00</td><td>1.0</td><td>99.0</td><td>0.600</td></tr>
</table>
* A = 70% ACN+30% H_2O /10 mM Ammonium Formate +0.1% Formic acid
** B = H_2O + 10 mM Ammonium Formate + 0.1% Formic Acid
</td></tr>
<tr><td>Stoptime</td><td>13 minutes</td></tr>
<tr><td>Temperature</td><td>5 °C</td></tr>
<tr><td>Injection Volume</td><td>5 µL</td></tr>
<tr><td>Flow</td><td>0.600 mL/min</td></tr>
<tr><th colspan="2">Q-TOF</th></tr>
<tr><td>Drying gas</td><td>8 L/min</td></tr>
<tr><td>Fragmentor</td><td>150 V</td></tr>
<tr><td>Vcap</td><td>3500 V</td></tr>
<tr><td>Ion polarity</td><td>Positive</td></tr>
</table>

**Mass range (m/z)**	100-1000
**Acquisition Rate**	2 spectra/s

S2. PEGDE quantification method details

The HPLC-QTOF measures were performed on an Agilent 1260 Infinity II HPLC system coupled to an Agilent 6530 Q-TOF mass spectrometer. Chromatographic separation was performed using Agilent InfinityLab Poroshell 120EC C18 column (150 x 3.0 mm, 2.7μm).

Before the starting of the measurements, the system tuning was performed with undiluted ESI-L Tune/Calibration mix TOF (G1969-85000) supplied by Agilent Technologies. Ions 121.050873. and 922.009798 were selected as reference ions during the measurements, coming from a reference solution prepared by diluting the G1969-85001 ES-TOF Reference Mass Solution Kit in acetonitrile/water according to Agilent instructions.

Table S2. HPLC-QTOF method details for method validation for PEGDE content in HA hydrogels.

<table>
<tr><td colspan="2">LC</td></tr>
<tr><td>Gradient</td><td>
<table>
<tr><th>Time (min)</th><th>A* (%)</th><th>B ** (%)</th><th>Flow (mL/min)</th></tr>
<tr><td>0.00</td><td>5.0</td><td>95.0</td><td>0.600</td></tr>
<tr><td>1.00</td><td>5.0</td><td>95.0</td><td>0.600</td></tr>
<tr><td>6.00</td><td>65.0</td><td>35.0</td><td>0.600</td></tr>
<tr><td>7.00</td><td>65.0</td><td>35.0</td><td>0.600</td></tr>
<tr><td>8.00</td><td>100.0</td><td>0</td><td>0.600</td></tr>
<tr><td>9.00</td><td>100.0</td><td>0</td><td>0.600</td></tr>
<tr><td>10.00</td><td>5.0</td><td>95.0</td><td>0.600</td></tr>
</table>
* A = 70% ACN+30% H_2O /5 mM Ammonium Formate +0.1% Formic acid
** B = H_2O + 5 mM Ammonium Formate + 0.1% Formic Acid
</td></tr>
<tr><td>Stoptime</td><td>13 minutes</td></tr>
<tr><td>Temperature</td><td>5 °C</td></tr>
<tr><td>Injection Volume</td><td>5 μL</td></tr>
<tr><td>Flow</td><td>0.600 mL/min</td></tr>
<tr><td colspan="2">Q-TOF</td></tr>
<tr><td>Drying gas</td><td>8 L/min</td></tr>
<tr><td>Fragmentor</td><td>150 V</td></tr>
<tr><td>Vcap</td><td>3500 V</td></tr>
<tr><td>Ion polarity</td><td>Positive</td></tr>
<tr><td>Mass range (m/z)</td><td>100-1000</td></tr>
</table>

**Acquisition Rate**	2 spectra/s

S3. PEGDE Certificate of Analysis

7/03/22, HC

BOC Sciences®

W: www.bocsci.com T: 1-631-504-6093 F: 1-631-614-7828 E: info@bocsci.com

Certificate of Analysis

(Poly(ethylene glycol) diglycidyl ether, Mn=500

CAS No.	72207-80-8	Batch No.	B20U06043
Synonyms	α,ω-Diglycidyl poly (ethylene glycol) - 500	Packing Quantity	10 g
TESTING ITEM	SPECIFICATION	RESULT	
Appearance	Colorless to yellowish liquid	Yellowish liquid	
Color	≤ 4	Conforms	
Identity (HNMR) *	Conforms to structure	Conforms	
Substitution (HPLC) *	≥ 93.0%	99.0%	
Average molecular weight *	500 ± 50 Da	534 Da	
Moisture (K.F.) *	≤ 2.0%	0.01%	
Residual solvents *	Dichloromethane ≤ 600 ppm	50 ppm	
	Tetrahydrofuran ≤ 720 ppm	50 ppm	
	Toluene ≤ 890 ppm	50 ppm	
	n-Heptane ≤ 5000 ppm	50 ppm	
Endotoxins *	≤ 0.1 EU/mg	0.1 EU/mg	
Conclusion	The product complies with the given specifications.		
Storage	- 20 °C		

QC: Harry Garrett **Approved by:** Zoey Tseng **Date:** *June 28, 2020*

BOC Sciences products are to be used for research purposes only. They may not be used for any other purpose, including, but not limited to, use in drugs, in vitro diagnostic purposes, therapeutics, or in humans unless an exception stated on the label or certificate. This document is not intended to assure certain properties of products or their suitability for a specific application.

BOCSCI Inc., 45-16 Ramsey Road, Shirley, NY 11967, USA

Figure S1. Py-GC-MS mass spectra comparison of (a) the peak at RT= 21.75 in the first shot and (b) the peak at RT=21.43 min in the second shot.

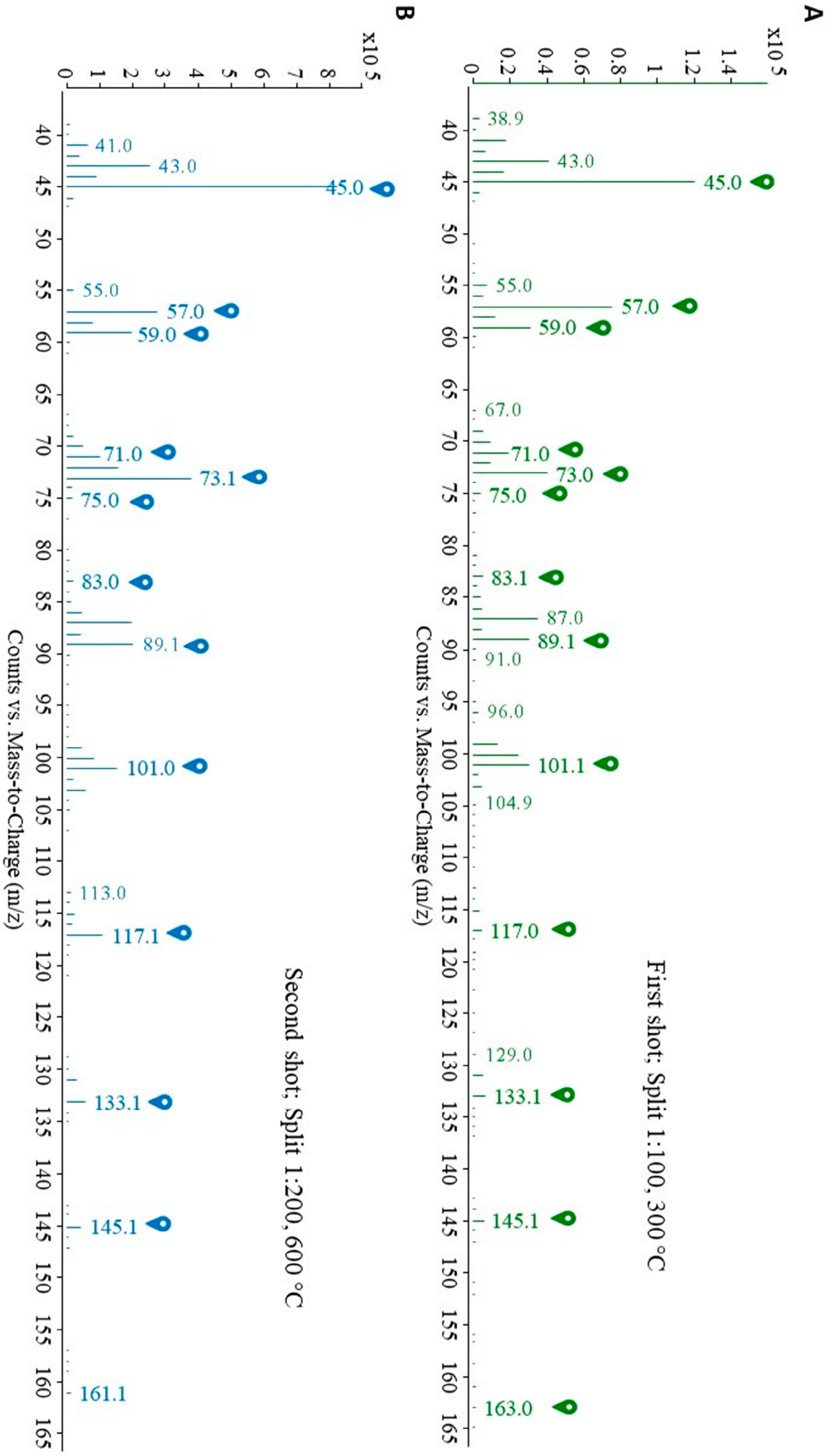

Figure S2. Fragment identification in Py-GC-MS mass spectra of the peak at RT= 21.75 in the first shot.

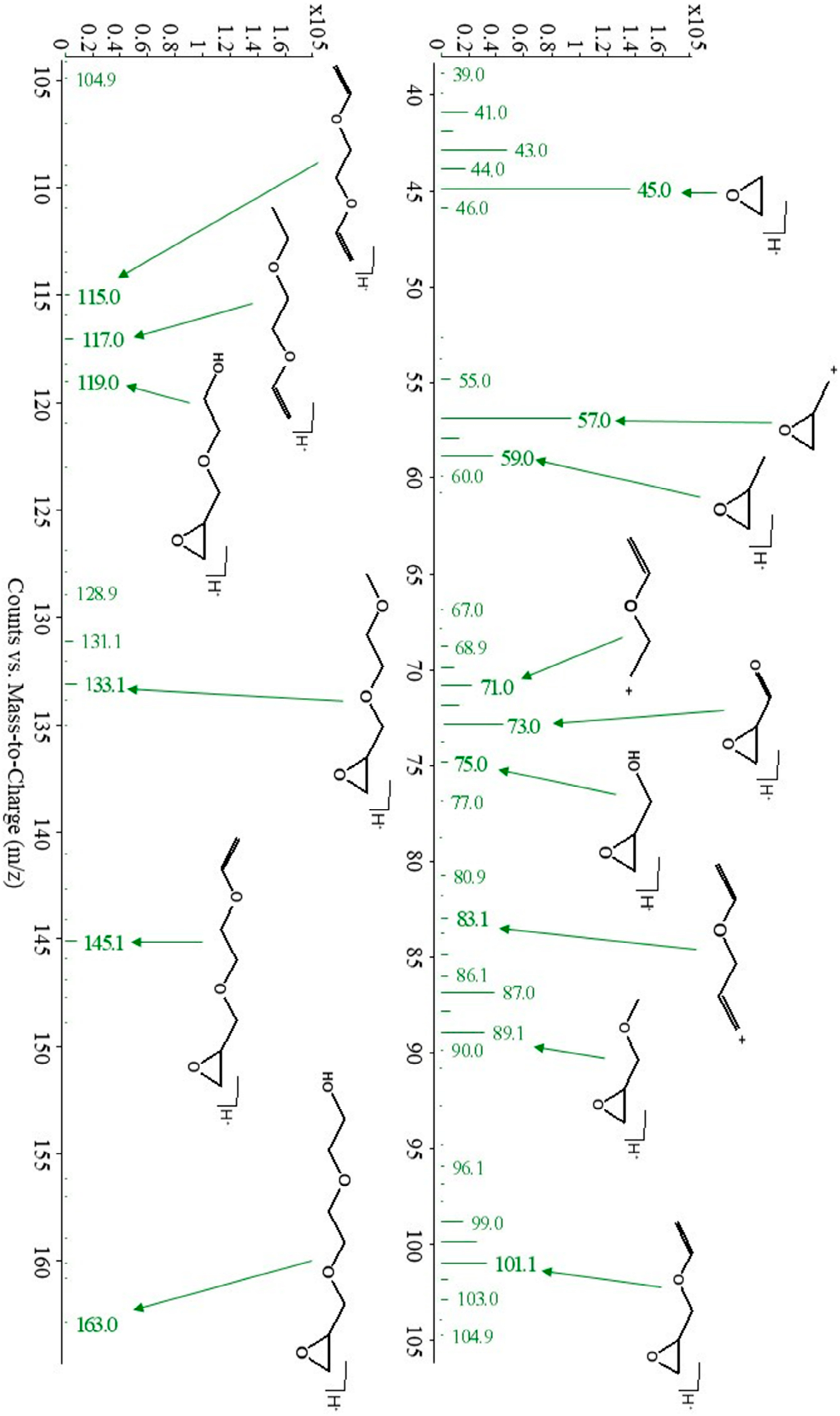

Figure S3. PEGDE fragmentation.

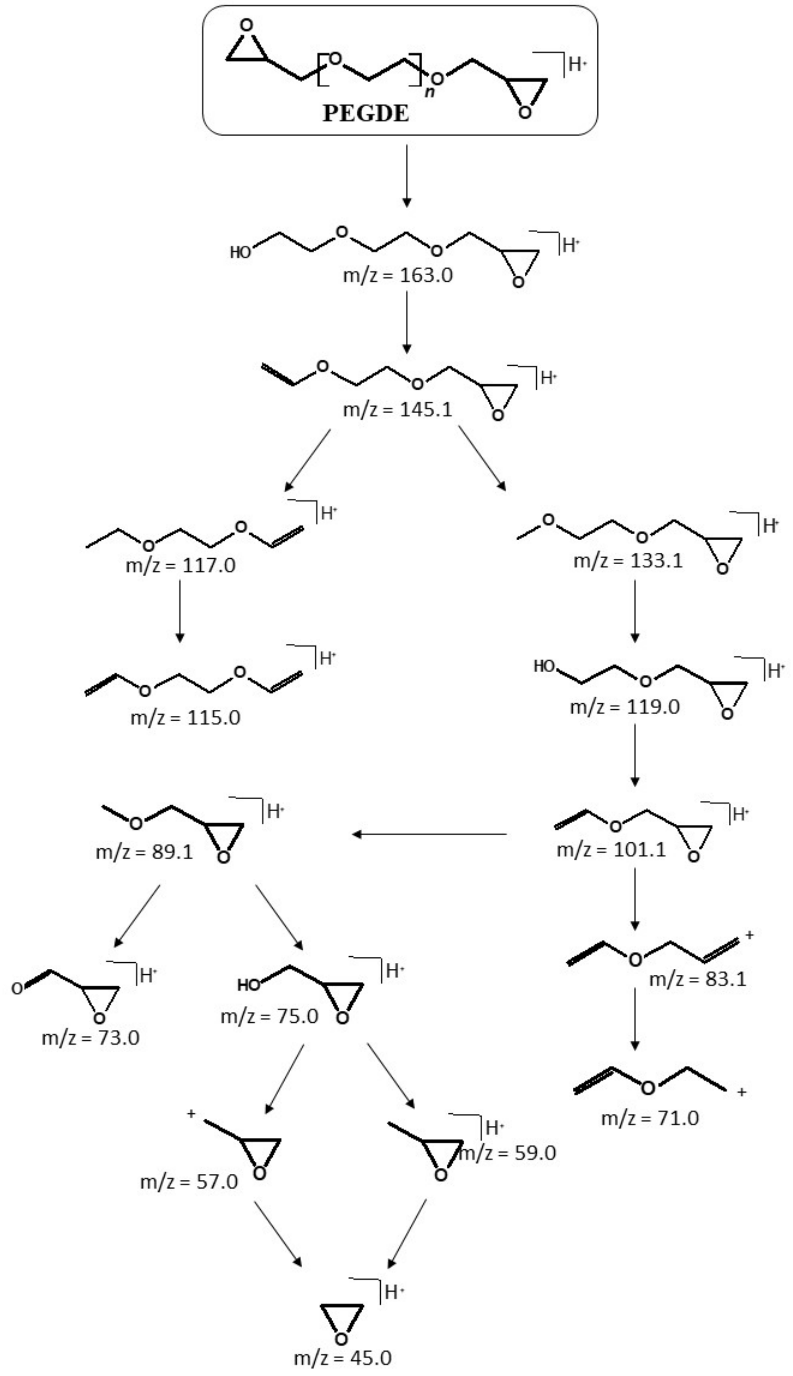

Supplement: Supplementary file 1 [file gels-09-00409-s001.zip › gels-2313286-supplementary.pdf]
